# Impact of hyaluronic acid filler on lip impressions: a cheiloscopic study

**DOI:** 10.1016/j.jobcr.2025.08.025

**Published:** 2025-08-27

**Authors:** Kananda Loiola Fernandes de Aguiar, Vandilson Pinheiro Rodrigues, Rafael Soares Diniz, Victor RM. Munoz-Lora, José Ferreira Costa, Letícia Machado Gonçalves-Soares

**Affiliations:** a Department of Dentistry, Federal University of Maranhão, São Luís, Maranhão, Brazil; b Postgraduate Program in Dentistry, Federal University of Maranhão, São Luís, Maranhão, Brazil; c GS Dentistry & Education, São Luís, Maranhão, Brazil; d Department of Periodontology an Implantology, University of Guarulhos, São Paulo, Brazil; e Let's HOF Academy, São Paulo, Brazil

**Keywords:** Hyaluronic acid, Lip prints, Dermal fillers, Legal dentistry

## Abstract

**Objectives:**

The objective of this study was to evaluate, through cheiloscopic analysis, the impact of hyaluronic acid (HA) lip augmentation on lip impressions. Eleven patients underwent HA injections and had their lips analyzed for thickness, morphological classification, commissure position, and lip impression types at three time points: before treatment (T1), one month after (T2), and three months after (T3). Lip thickness was measured using a digital caliper, and commissure position was classified based on standardized digital photographs. For lip impression recording, lipstick was applied, and the lips were then pressed against a substrate. The types of lip grooves were determined after dividing the impression into eight quadrants. Data were statistically analyzed using repeated measures ANOVA and Fisher's exact test, with a significance level of 5 %.

**Results:**

There was a significant increase in both upper and lower lip thickness, which remained stable after 3 months (*p* < 0,001). Regarding morphological classification, medium and mixed lips maintained their pattern in most cases, while thin lips became medium or mixed. No patient had “thick” or “very thick” lips after the treatment. There were also important changes in commissure position, particularly among patients with “lowered” commissures, which shifted to “horizontal.” No variations in lip impressions were observed across the quadrants examined at any time point.

**Conclusion:**

The findings of this study suggest that, for purposes of human identification, the pattern of lip impressions remains unchanged after HA lip augmentation.

## 1 Introduction

Forensic experts play a critical role in human identification through techniques such as the analysis of bite mark and lip print analyses, dental arch comparisons in cases of charred or decomposed bodies, and skeletal examination in forensic anthropology.^1^ In criminal investigations, identification methods must rely on comparative data analysis and adhere to essential principles — including immutability, individuality, perenniality, classifiability, and practicability — to be deemed scientifically reliable.^1^, ^2^, ^3^, ^4^

The most commonly used methods in forensic investigations are fingerprint analysis, dental records, and DNA testing. However, in some cases these methods may not be applicable, prompting professionals to employ lesser known but valid techniques, such as cheiloscopy. This technique focuses on examining lip anatomy and structure, which can play a crucial role in identifying or ruling out potential suspects.^3^^,^^4^

Similar to fingerprint analysis, lip prints can be used to identify individuals based on marks left at crime scenes.^5^ Cheiloscopic analysis examines the fine grooves on the labial mucosa, which form unique and individualized patterns — even among monozygotic twins.^6^, ^7^, ^8^, ^9^ These impressions are considered immutable, as injuries typically heal without altering their original structure,^10^ and they remain stable over time, ensuring perenniality.^11^ Their low-cost, ease of collection, and classification potential further support their forensic applicability.^12^

Today, growing concerns about aesthetic standards have significantly influenced social well-being, with self-perception, public opinion, and social acceptance playing a major role. This trend, amplified by social media culture, has led to a surge in demand for dermatological and dental aesthetic procedures. One of the most sought-after treatments is lip augmentation with hyaluronic acid (HA), which enhances lip volume, reshapes lip anatomy, and promotes deep tissue hydration and ranking as the second most commonly performed non-surgical facial aesthetic treatment in the United States.^13^

With the increasing popularity of lip augmentation with HA, questions have arisen in forensic studies regarding its potential impact on lip impression patterns. Since the procedure alters lip shape, thickness, and width, it is reasonable to suspect that it may also affect lip impressions. However, existing literature has yet to determine whether the deep hydration provided by HA can alter lip print patterns, as the product is designed to smooth out furrows and fine wrinkles. This question is of critical forensic importance, as any alteration to lip prints could challenge the reliability of cheiloscopy and introduce uncertainty in suspect identification or elimination.

Therefore, the objective of this study was to evaluate, through cheiloscopic analysis, the effect of HA lip augmentation on lip impressions and to determine whether this method continues to uphold the fundamental principles required for human identification.

## 2 Methods

### 2.1 Study design

This study was designed as a prospective longitudinal analysis of lip augmentation procedures conducted between January and July 2024 at the GS Odontologia & Ensino Institute (São Luis, Maranhão). The study received ethical approval from the Research Ethics Committee of the University Hospital HUUFMA under the protocol number CAAE – 57656222.7.0000.5086. All participants provided written informed consent prior to their inclusion, authorizing the use of their clinical and photographic data for research purposes. The aesthetic procedures performed (lip augmentation using HA-based fillers) followed established clinical protocols and were conducted in accordance with Good Clinical Practice guidelines.

### 2.2 Volunteers

Eleven (n = 11) consecutive, multi-ethnic Brazilian female patients aged between 20 and 40 years who were seeking for lip augmentation were included. Inclusion criteria were limited to female patients without pre-existing lip malformations, trauma, infections, or inflammation in the perioral region. Exclusion criteria included previous lip augmentation with either temporary or permanent fillers, any other aesthetic treatment of the lips, or any systemic condition that could interfere with healing or tissue response. The primary outcome of this study was to evaluate changes in lip print patterns at 90 days following treatment; the 30-day follow-up served as an intermediate evaluation point.

### 2.3 Lip augmentation technique

All patients were prepared by thoroughly washing their faces with water and neutral soap, followed by asepsis using 2 % aqueous chlorhexidine and a mouthwash with 0.2 % chlorhexidine. All procedures were performed by the same clinician with more than 5 years of experience, using the exact same standardized injection technique described below.

Anesthesia was administered through extraoral nerve blocks targeting the infraorbital, buccal, and mental nerves, using 3 % mepivacaine without vasoconstrictor (DFL Indústria e Comércio SA, Rio de Janeiro, Brazil). A HA filler, specifically formulated for lip enhancement (Restylane Kysse, Galderma, Texas, USA), with a concentration of 20 mg/mL was used for lip augmentation.

For lip filling, a cannula entry point was established at the highest portion of the cupid's bow for the upper lip on each hemilabium, and in the corresponding position on the lower lip, using a 21G needle (Terumo, São Paulo, Brazil). Subsequently, a 22G cannula (Terumo, São Paulo, Brazil) was used for linear retroinjection of 0.20 mL bilaterally in the submucosal plane, for the upper lip, and 0.30 mL bilaterally for the lower lip. No manipulation or massage was performed after the procedure.

After the procedure, patients were given verbal and written postoperative instructions, including information on expected swelling, inflammation, and proper care.

### 2.4 Outcomes

Three primary outcomes were evaluated in this study: lip thickness and morphological classification, position of the lip commissures, and cheiloscopic groove patterns. These parameters were assessed at three time points: prior to lip augmentation (T1), 1 month after the procedure (T2), and 3 months after the procedure (T3).

All analyses were conducted by the same examiner, who had been previously calibrated by an experienced forensic dentistry specialist. Intra-examiner reliability was confirmed through a pilot study with three volunteers. The examiner classified groove types at two separate time points, one week apart, under identical environmental conditions. A Kappa coefficient greater than 0.81 was required to confirm consistency.

### 2.5 Labial thickness and morphological classification

Labial thickness was assessed using a digital caliper (Mitutoyo, São Paulo, Brazil). For the upper lip, the measurement was taken from the apex of the cupid's bow to the wet-dry border. For the lower lip, it was measured from the wet-dry border to the central portion of the vermillion margin. All values were recorded in millimeters, and the arithmetic mean was calculated for each lip.

In addition to objective measurements, lips were morphologically classified according to the criteria proposed by Santos et al.,^15^ as follows:

• *Thin:* narrow lips, typically associated with individuals of Caucasoid ancestry.
• *Medium:* lips with a rounded mucosa, ranging from 8 to 10 mm in thickness.
• *Thick or Very Thick:* prominent, voluminous lips with evident eversion and white lines, often seen in individuals of African descent.
• *Mixed:* combination of a thin upper lip and a thick lower lip, commonly observed in individuals of East Asian ancestry.

### 2.6 Lip commissure position assessment

The position of the lip commissures was evaluated using standardized digital photographs taken with a Canon T7i camera equipped with a 100 mm macro lens. All images were taken under natural lighting conditions, at a fixed distance of 1 m, with patients in a relaxed position at rest.

The commissures were classified into three categories, as described by Muñoz:^16^

• *Lowered:* positioned below the imaginary horizontal line drawn tangent to the labial tubercle.
• *Horizontal:* aligned with the tangent line.
• *Elevated:* positioned above the tangent line.

### 2.7 Labial grooves classification

Lip impressions were obtained using a layer of long-lasting red lipstick (Mary Kay, Texas, USA) applied to clean, dry lips. Patients gently pressed their closed lips against a white cardboard sheet placed on a glass surface, performing a controlled side-to-side rolling motion. The impressions were immediately sealed with 48-mm-wide transparent adhesive tape (3M) to preserve detail. All samples were coded and cataloged along with basic demographic data and self-declared ethnicity.

**Fig. 1:**
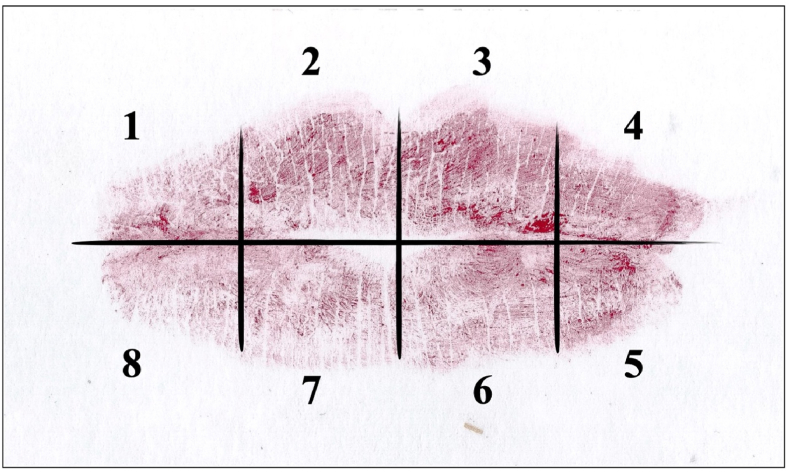
Schematic representation of the eight quadrants analyzed in the lip impression, numbered clockwise for cheiloscopic evaluation.

**Fig. 2:**
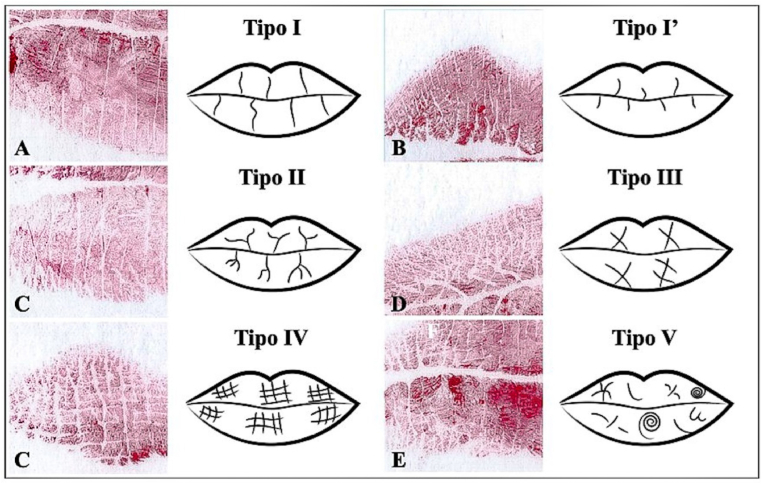
Classification of labial groove patterns: (A) Type I – complete vertical lines; (B) Type I′ – incomplete vertical lines; (C) Type II – branched or bifurcated lines; (D) Type III – crossed lines; (E) Type IV – reticulated lines; (F) Type V – irregular or unclassifiable lines.

Cheiloscopic patterns were analyzed following the methodology of Suzuki and Tsuchihashi.^17^ Each impression was divided into eight quadrants, numbered clockwise (Fig. 1). The dominant groove type in each quadrant was identified using a magnifying glass and recorded in a standardized cheilogram. The grooves were classified into the following types (Fig. 2):

• Type I: complete vertical lines
• Type I′: incomplete vertical lines
• Type II: branched or bifurcated lines
• Type III: crossed lines (X- or quotation-mark-shaped)
• Type IV: reticulated lines (net-like appearance)
• Type V: grooves that do not fit into the previous categories

### 2.8 Statistical analysis

Statistical analyses were conducted using GraphPad Prism software (version 9.0, GraphPad Software, San Diego, CA, USA), with the significance level set at p ≤ 0.05. Descriptive statistics were initially performed, with quantitative variables presented as the mean and standard deviation (SD), and categorical variables expressed as absolute and relative frequencies (%).

To assess differences in lip thickness across the three time points (T1 – baseline, T2 – 1 month, T3 – 3 months), a repeated measures ANOVA was applied, followed by Tukey's post-hoc test for multiple comparisons. For categorical variables, including commissure position and labial groove types, Fisher's exact test was used to evaluate changes over time.

## 3 Results

### 3.1 Demographics

This prospective longitudinal study included 11 female patients with a Brazilian multi-ethnic background, aged between 20 and 40 years, with a mean age of 29.6 (±5.6) years at baseline. Self-reported ethnic distribution was as follows: 27.3 % identified as brown, 45.5 % as white, and 27.3 % as yellow; no participants identified as black or indigenous.

All participants met the eligibility criteria and completed the three time-point evaluations (T1, T2, and T3). No statistically significant demographic differences were observed that might impact the primary outcomes. For detailed demographic information, see Table 1.

**Table 1:** Demographic data showing quantitative measurements of lip thickness (mm), corresponding morphological lip classification, and lip commissure position at each time point.

Case	Lip thickness (mm)						Lip morphology			Commissure position		
	Upper lip			Lower lip								
	T1	T2	T3	T1	T2	T3	T1	T2	T3	T1	T2	T3
**1**	7	9	9	9	11	11	Mixed	Mixed	Mixed	L	H	H
**2**	7	9	9	10	11	11	Mixed	Mixed	Mixed	L	H	H
**3**	7	9	9	7	9	9	Thin	Medium	Medium	L	H	H
**4**	10	10	10	7	10	10	Mixed	Medium	Medium	H	E	E
**5**	8	9	9	8	10	10	Medium	Medium	Medium	L	H	H
**6**	8	10	10	7	9	9	Mixed	Medium	Medium	E	E	E
**7**	7	8	8	7	8	8	Thin	Medium	Medium	H	E	E
**8**	8	10	10	11	12	12	Mixed	Mixed	Mixed	H	E	E
**9**	8	9	9	10	11	11	Medium	Mixed	Mixed	H	E	E
**10**	6	8	8	7	9	9	Thin	Medium	Medium	H	E	E
**11**	5	7	7	9	11	11	Mixed	Mixed	Mixed	L	H	H

L = lowered. H = horizontal. E = elevated.

### 3.2 Lip thickness

A statistically significant increase in both upper and lower lip thickness was observed following HA filler injection. The mean upper lip thickness increased from 7.3 (±1.2) mm at baseline (T1) to 8.9 (±0.9) mm at both 1 month (T2) and 3 months (T3) post-treatment (*p* < 0.001). Similarly, the lower lip thickness increased from 8.3 (±1.5) mm at T1 to 10.1 (±1.2) mm at T2 and remained stable at T3 (*p* < 0.001). No significant differences were detected between T2 and T3 for either lip (*p* = 1.000), indicating volumetric stability during the three-month follow-up. Detailed results are presented in Table 2.

**Table 2:** Comparative analysis of upper and lower lip thickness (mm) across the three evaluation time points: T1 (baseline), T2 (1-month post-treatment), and T3 (3-months post-treatment).

Lip	Lip thickness (mm)			T2 *vs* T1	T3 *vs* T1	T3 *vs* T2
	(T1)	(T2)	(T3)	Δ *P*	Δ *P*	Δ *P*
	mean ± SD	mean ± SD	mean ± SD			
Upper lip	7.3 ± 1.2	8.9 ± 0.9	8.9 ± 0.9	+1.55 ***P* < 0.001**	+1.55 ***P* < 0.001**	ND p = 1.000
Lower lip	8.3 ± 1.5	10.1 ± 1.2	10.1 ± 1.2	+1.73 ***P* < 0.001**	+1.73 ***P* < 0.001**	ND p = 1.000

Values are expressed as mean ± standard deviation (SD). Δ = mean difference between time points. ND = no difference detected. Statistical analysis performed using repeated measures ANOVA with Tukey's post-hoc test. Significant results are marked in bold (*P* < 0.05).

### 3.3 Morphological lip classification

At baseline (T1), lip morphology was classified as thin in 3 patients (27.3 %), medium in 3 patients (27.3 %), and mixed in 5 patients (45.5 %). After treatment, all lips initially classified as thin transitioned to either medium or mixed morphology. Patients with medium or mixed lips at baseline maintained their classification at T2 and T3. No cases were classified as “thick” or “very thick” at any evaluation time point, reflecting the natural volumizing profile of the HA filler used. Individual results for each patient are presented in Table 1.

### 3.4 Lip commissure position

Significant changes were observed in the classification of lip commissure position over time (p = 0.011). At baseline (T1), 5 patients (45.5 %) presented with “lowered” commissures; by both T2 and T3, none remained in this category. Instead, these patients transitioned to “horizontal” or “elevated” positions. The number of patients with “elevated” commissures increased from 1 (9.0 %) at baseline to 6 (54.5 %) at both post-treatment evaluations. The “horizontal” classification remained unchanged in 5 patients (45.5 %) throughout the study. Detailed results are provided in Table 3.

**Table 3:** Distribution of lip commissure classifications at each time point: T1 (baseline), T2 (1-month post-treatment), and T3 (3 months post-treatment).

Lip commissure classification	T1	T2	T3	*P*-Value
	n (%)	n (%)	n (%)	
Lowered	5 (45,5)	0 (0)	0 (0)	0.011∗
Horizontal	5 (45.5)	5 (45.5)	5 (45.5)	
Elevated	1 (9.0)	6 (54.5)	6 (54.5)	

Values are expressed as absolute frequencies (n) and percentages (%). Statistical analysis was performed using Fisher's exact test. (∗) = *P* < 0.05.

### 3.5 Cheiloscopic analysis

**Fig. 3:**
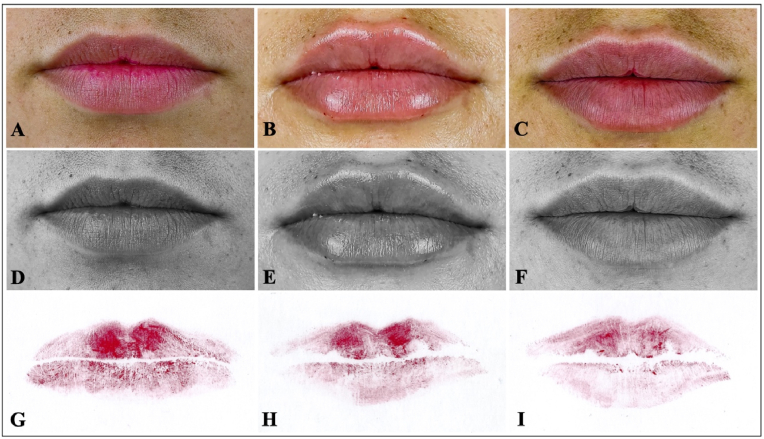
Photographic documentation of 22 years female participant. Color images of the lips at rest: (A) baseline (T1), (B) 1 month after filler (T2), and (C) 3 months after filler (T3). Corresponding black and white images for enhanced contrast: (D) T1, (E) T2, (F) T3. Cheiloscopic lip impressions collected at each time point: (G) T1, (H) T2, (I) T3.

**Fig. 4:**
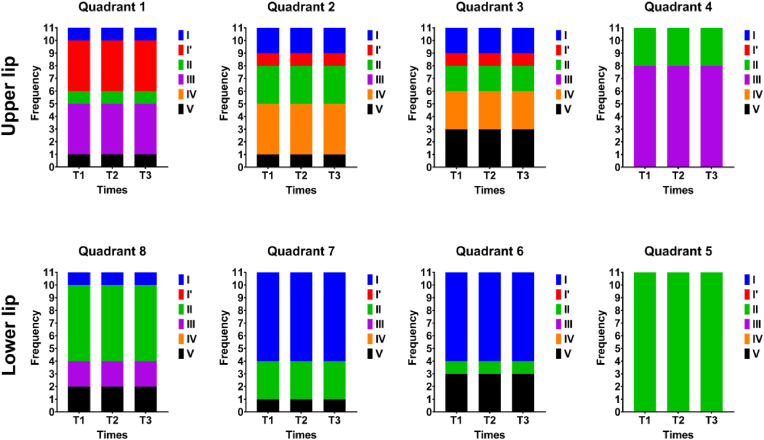
Comparative analysis of labial groove classifications across the eight quadrants at the three evaluation time points (T1 – baseline, T2 – 1 month post-treatment, T3 – 3 months post-treatment).

Despite the volumetric and positional changes observed in the lips after filler application, cheiloscopic analysis revealed no changes in lip groove patterns (Fig. 3). Across the eight quadrants examined per patient, the groove types remained identical between T1, T2, and T3. The dominant groove type in each quadrant—classified as Type I, I′, II, III, IV, or V—did not vary over time in any patient. These findings indicatea strong stability of cheiloscopic patterns following HA augmentation. The full quadrant-by-quadrant classification is presented in Table 4, Table 5, and the overall comparison is illustrated in Fig. 4.

**Table 4:** Individualized cheiloscopic classification of the upper lip across four quadrants at the three time points: T1 (baseline), T2 (1-month post-treatment), and T3 (3 months post-treatment).

Case	Quadrant 1			Quadrant 2			Quadrant 3			Quadrant 4		
	T1	T2	T3	T1	T2	T3	T1	T2	T3	T1	T2	T3
**1**	V	V	V	I	I	I	I	I	I	III	III	III
**2**	III	III	III	II	II	II	II	II	II	III	III	III
**3**	II	II	II	IV	IV	IV	IV	IV	IV	II	II	II
**4**	III	III	III	II	II	II	I'	I'	I'	III	III	III
**5**	III	III	III	I	I	I	V	V	V	III	III	III
**6**	III	III	III	I'	I'	I'	I	I	I	II	II	II
**7**	I'	I'	I'	IV	IV	IV	IV	IV	IV	II	II	II
**8**	I'	I'	I'	IV	IV	IV	IV	IV	IV	III	III	III
**9**	I'	I'	I'	II	II	II	V	V	V	III	III	III
**10**	I	I	I	V	V	V	V	V	V	III	III	III
**11**	I'	I'	I'	IV	IV	IV	II	II	II	III	III	III

**Table 5:** Individualized cheiloscopic classification of the lower lip across four quadrants at the three time points: T1 (baseline), T2 (1-month post-treatment), and T3 (3 months post-treatment).

Case	Quadrant 5			Quadrant 6			Quadrant 7			Quadrant 8		
	T1	T2	T3	T1	T2	T3	T1	T2	T3	T1	T2	T3
**1**	II	II	II	I	I	I	I	I	I	II	II	II
**2**	II	II	II	I	I	I	I	I	I	II	II	II
**3**	II	II	II	I	I	I	I	I	I	V	V	V
**4**	II	II	II	V	V	V	I	I	I	I	I	I
**5**	II	II	II	I	I	I	II	II	II	II	II	II
**6**	II	II	II	II	II	II	I	I	I	V	V	V
**7**	II	II	II	I	I	I	I	I	I	III	III	III
**8**	II	II	II	I	I	I	V	V	V	II	II	II
**9**	II	II	II	I	I	I	II	II	II	II	II	II
**10**	II	II	II	V	V	V	I	I	I	II	II	II
**11**	II	II	II	V	V	V	II	II	II	III	III	III

## 4 Discussion

Currently, society is increasingly inclined toward facial aesthetic procedures, with lip fillers among the most sought-after treatments. Consequently, questions arise about forensic investigation techniques, such as cheiloscopy since any significant interference in the pattern of lip impressions could challenge the method's reliability and even cause doubts in the identification and/or elimination of a possible criminal suspect. The novelty of this study lies in the fact that, for the first time in the scientific literature, the impact of HA fillers on lip prints has been evaluated using cheiloscopic analysis. The main findings indicate an increase in lip thickness and a change in the position of the lip commissures; however, the pattern of lip impressions remained unchanged. These results reinforce the potential applicability of lip prints as a reliable method for human identification, even after undergoing this type of cosmetic procedure.

It is known that lip filling with HA is a technique employed with efficacy, safety, as well as a high rate of satisfaction by the patients.^18^^,^^19^ A recent clinical study investigated the clinical and histological effects of lip augmentation, showing no inflammatory response and stable filler volume at 30 days. After 60 days, the first signs of resorption of the filler began, with few infiltrated inflammatory cells.^18^ Considering these results, the study design defined a follow-up time of three months, as it was expected that possible changes in lip impressions would occur in the first months and might remain or not as the HA underwent its resorption process.

Only women were included in this study, given the well-documented anatomical differences between male and female lips. Men generally have thinner lips compared to women, who tend to have more voluminous lips and greater vermilion height. Such peculiarities demonstrate that the operative approach should be distinct to ensure more effective and satisfactory results.^20^ Therefore, to standardize the filling technique and minimize biases, we chose to include only women in the study.

In cheiloscopic studies, the examiner must have both theoretical and practical expertise in the technique to ensure accurate classification. This process can be challenging, as grooves may overlap or coexist in different lip regions. Therefore, calibration is essential to achieve a high replicability in the evaluation method. It is important to emphasize that calibration should be performed both by a single examiner and between different examiners, as occurs in epidemiological surveys, in which the demand for surveyed volunteers requires a larger number of research participants.^14^^,^^21^ In this study, a single calibrated examiner conducted all assessments, making only intra-examiner calibration necessary.

The market offers numerous types and commercial brands of HA-based filler gels, which may differ in duration of effect, AH concentration, rheology, adverse event profiles, and ease of use.^22^ For this study, a filler developed specifically for the lip region (Restylane Kysse, Galderma, Texas, USA) was used. It contains 20 mg/mL of HA, has a mild-to-moderate gel texture, moderate hydration capacity, low volumizing power, and moderate-to-low lifting ability. According to the manufacturer, it is recommended for lip projection, volumizing, eversion, and contouring, with a focus on achieving natural results.

After the filling, a moderate increase in lip thickness was observed, confirming the more natural effects promised by the product. As for lip thickness, the patients who sought treatment had a mixed, thin and medium pattern. After the filling, the mixed and medium lips maintained their pattern, while the thin lips became medium or mixed. No patient presented “thick” or “very thick” lips after the application. Regarding thickness, there was an increase in both upper and lower lips, which remained stable after the 3-month analysis.

Significant changes were also noted in commissure position, particularly in patients initially presenting with “lowered” commissures, which shifted to a “horizontal” position. These outcomes were expected, since lip filling aims to improve shape, volume, and reduce wrinkles. Furthermore, these changes remained stable over the 90-day evaluation period. It is important to highlight that even though these changes occurred, these parameters (thickness and position of the commissures) are complementary, but not decisive in forensic interpretation.^15^^,^^16^

In forensic analysis, the decisive parameter is the lip print itself. Identification depends on the presence of coinciding points between the recorded lip grooves and the reference print.^8^^,^^21^ For the comparison between the three time points to be considered positive, at least 12 convergent points (the same criterion used for fingerprints) had to be identified through direct comparison, placing the three prints side by side and enumerating homologous points.^21^^,^^23^

Like fingerprints, lip prints are unique and can confirm an individual identity. Fingerprints may be altered by abrasions, deep cuts, burns, chemical exposure, or certain dermatological diseases that affect papillary ridges.^24^ For lip impressions, our results showed no changes in groove patterns over time. Even though HA is intended to hydrate and smooth wrinkles, it did not interfere with cheiloscopic identification. These results indicate that hyaluronic acid lip filling does not alter the cheiloscopic pattern, supporting its characteristics of perenniality, individuality, and immutability.

Although cheiloscopy is a validated forensic method, its use should be prioritized only when traditional identification methods are unavailable.^3^^,^^4^ It is not uncommon to find latent or visible lip prints on glasses, paper, cups, napkins, or cigarettes at crime scenes.^25^ Nevertheless, some authors consider lip prints less reliable than fingerprints for human identification.^26^^,^^27^

This study has several strengths, including the longitudinal follow-up, standardized procedure and product, and the use of examiner calibration with high intra-examiner agreement. However, limitations should be noted: the small sample size, despite a high effect size, inclusion of only female participants, and exclusion of certain ethnic groups may reduce the external validity of the findings. Future studies with larger, more diverse samples, varying filler types and volumes, and inclusion of male participants are recommended to expand understanding of these variables in different clinical contexts.

## 5 Conclusion

Within the limitations of this study, the findings suggest that, for purposes of human identification, the pattern of lip impressions remains unchanged after hyaluronic acid lip augmentation.

## Patient's consent

The study received ethical approval from the Research Ethics Committee of the University Hospital HUUFMA under the protocol number CAAE – 57656222.7.0000.5086. All participants provided written informed consent prior to their inclusion, authorizing the use of their clinical and photographic data for research purposes. The aesthetic procedures performed (lip augmentation using hyaluronic acid fillers) followed established clinical protocols and were conducted in accordance with Good Clinical Practice guidelines.

## Disclosures

The authors have no proprietary, financial, or other personal interest of any nature or kind in any product, service, and/or company that is presented in this article.

## Sources of funding

The authors received no financial support for the research, authorship, and publication of this article. The authors have no proprietary, financial, or other personal interest of any nature or kind in any product, service, and/or company that is presented in this article.

## Declaration of competing interest

The authors declare that they have no known competing financial interests or personal relationships that could have appeared to influence the work reported in this paper.
